# Editorial: Sexual abuse and women's mental health

**DOI:** 10.3389/fpsyg.2024.1491803

**Published:** 2024-10-11

**Authors:** Sherry H. Stewart, Alessandra M. Passarotti

**Affiliations:** ^1^Department of Psychiatry, Dalhousie University, Halifax, NS, Canada; ^2^Mood, Anxiety, and Addiction Comorbidity (MAAC) Lab, Department of Psychology and Neuroscience, Dalhousie University, Halifax, NS, Canada; ^3^Department of Psychology, University of Illinois Chicago, Chicago, IL, United States

**Keywords:** sexual abuse, sexual violence, sexual assault, sexual victimization, mental health, addictive behavior, women, gender

This Research Topic delves into the intricate relationships between sexual victimization (SV) and women's mental health and addictive behaviors across the lifespan. This Research Topic brings together insights from experts in psychology and related fields, shedding light on the multifaceted impact of SV on women's wellbeing. By examining the intersection between SV and various forms of psychopathology such as posttraumatic stress disorder (PTSD), borderline personality disorder (BPD), sexual concerns, anxiety, depression, and addictive behaviors/disorders, this Research Topic fosters a deeper understanding of the psychological, emotional, and behavioral consequences of SV in women.

This Research Topic comprises five articles from international teams, each primarily or jointly first-authored by a woman. Several feature the work of women early-career researchers together with senior women colleagues. We review some of the main contributions of each article. All are mechanistic studies focused on increasing understanding of the adverse emotional and/or behavioral consequences of SV—including childhood sexual abuse (CSA) and sexual assault (SA) in adulthood. By identifying potential mediators and moderators in these SV-to-psychopathology links, these articles suggest intervention targets for improved outcomes. They also identify which subpopulations are particularly vulnerable and most in need of intervention.

Zheng et al. examined a serial mediation model to elucidate sequential processes that help explain the strong link of CSA to depressive symptoms in emerging adult women. CSA was associated with holding negative self-schemas (e.g., “I am unlovable”) which in turn were associated with using experiential avoidance as a short-term coping strategy that ultimately exacerbates depressive symptoms in the longer-term. Results point to two important intervention targets for preventing/treating depressive symptoms in women CSA survivors: “negative core schema” can be targeted using Cognitive Behavioral Therapy techniques (Beck, 1991) and “experiential avoidance” can be targeted using Acceptance and Commitment Therapy strategies (Hayes et al., 2011). This cross-sectional study should be extended longitudinally to test the temporal ordering of variables in the sequential model.

Wais et al. also focused on the mental health consequences of CSA but among a sample of female adolescent inpatients. They studied a trauma symptoms composite as the outcome—i.e., PTSD, BPD, and sexual concern symptoms—focusing on the protective role of mentalizing (i.e., understanding reactions in psychological terms). CSA was positively related to the trauma symptoms composite, whereas mentalizing was negatively related (i.e., protective). Results suggest the potential utility of Mentalization-based Treatment (Fonagy and Target, 2006) for improving adolescent girls' resilience against various trauma sequelae. Future research might examine if mentalizing buffers against/moderates the adverse effects of CSA on trauma-related symptoms.

Balters et al. investigated the link between PTSD symptoms and alcohol misuse in emerging adult women. Daily diary methods were used to investigate the temporal dynamics of day-to-day variations in PTSD symptom networks (i.e., correlated sets of PTSD symptoms) and alcohol cravings/alcohol use. Three groups of heavy-drinking university women participated—SV history plus PTSD; SV history alone; and no SV history—allowing parsing of SV vs. PTSD effects. Women with PTSD showed greater temporal stability in PTSD symptom networks and arousal but greater variability in negative emotions and the two alcohol indices, suggesting women with PTSD may crave and use alcohol to cope with persistent PTSD symptoms, persistent arousal, and fluctuating negative mood states. PTSD symptom networks optimally predicted the two alcohol outcomes with a several-day time lag challenging self-medication theory predictions that PTSD symptoms should affect alcohol cravings/drinking behavior more immediately. Additionally, the predictive PTSD symptom networks involved primarily cognitive re-experiencing symptoms (e.g., intrusive memories). Findings suggest the need to develop therapeutic approaches that effectively target longer-range effects of PTSD symptoms, particularly re-experiencing, on alcohol craving/drinking behavior in university women.

Stewart, Strickland et al. examined processes explaining links of alcohol-involved sexual assault (AISA; i.e., SA when the victim was drinking) with negative emotional outcomes (depression, anxiety, and PTSD). While women were more likely to report AISA exposure and higher levels of anxiety and self-coldness, men reported greater fear of self-compassion. Shame was a significant mediator between AISA and all outcomes, suggesting the importance of targeting shame. Additionally, characterological self-blame mediated AISA's link with depressive symptoms, self-coldness mediated AISA's link with anxiety symptoms, and fear of self-compassion mediated AISA's link with PTSD symptoms. Thus, internalized stigma-based mechanisms are important intervention targets for young adult AISA survivors to prevent/treat emotional disorder symptoms.

Finally, Stewart, Khoury et al. examined relations of SA exposure to emotional and cannabis use outcomes. They examined whether SA has particularly adverse effects compared to other forms of trauma on emotional (depressive and PTSD symptom domains) and cannabis outcomes and whether there are gender differences in these links. Women reported higher rates of SA and greater use of cannabis to cope with negative emotions, suggesting cannabis coping motives (Simons et al., 1998) as an important treatment target for women cannabis users with trauma histories. Participants with SA histories scored higher than survivors of other traumas on several emotional and cannabis use outcomes, supporting SA as a particularly devastating form of trauma. They also found gender differences in the links of SA with certain emotional and cannabis outcomes. First, among SA survivors only, women scored higher than men in enhancement cannabis motives and purposeful cannabis craving, which may represent self-medication of women SA survivors' greater anhedonia via cannabis' euphoric effects. Second, SA survivors reported more PTSD negative mood/cognition symptoms than other trauma survivors, but only among men. The development of maladaptive beliefs (e.g., self-blame) and emotions (e.g., guilt) following SA vs. other traumas may be particularly likely among men survivors, given the heightened social stigma toward men SA survivors and/or the reluctance of men to disclose SA experiences. Future research should study the mechanisms underlying these gender moderation effects to determine if women and men SA survivors have different intervention needs.

Overall, this Research Topic educates about the mental health and substance use health needs of women who have experienced SV and identifies when women survivors' needs may be different than men's. By increasing awareness and understanding, this Research Topic may help create more inclusive and effective services that promote survivors' mental wellbeing.
